# Non-Digestible Oligosaccharides and Constipation: A Systematic Review and Meta-Analysis of Randomized Trials on Stool Frequency, Stool Consistency, and Fermentation Biomarkers

**DOI:** 10.3390/nu17203246

**Published:** 2025-10-16

**Authors:** Huiyu Chen, Jiale Ren, Langrun Wang, Wenyi Zhang, Sufang Duan, Jie Guo, Qingshan Chen, Ran Wang, Jian He, Jingjing He, Ruixin Zhu

**Affiliations:** 1 Key Laboratory of Precision Nutrition and Food Quality, Department of Nutrition and Health, China Agricultural University, Beijing 100083, China; 2 School of Public Health, Capital Medical University, Beijing 100069, China; 3 National Technology Innovation Center for Dairy, Hohhot 010100, Chinahejian@yili.com (J.H.); 4 Research Center for Probiotics, China Agricultural University, Beijing 100083, China

**Keywords:** non-digestible oligosaccharides (NDOs), functional constipation, stool frequency, Bristol stool scale, short-chain fatty acids

## Abstract

**Background**: Chronic constipation lacks effective long-term treatments. Non-digestible oligosaccharides (NDOs) are short-chain carbohydrates that resist digestion and may improve bowel function. This systematic review and meta-analysis examines the effect of NDOs on constipation-related outcomes in humans. **Methods**: We searched Ovid MEDLINE, Embase, and Web of Science (2010–May 2025) for randomized controlled trials (RCTs) comparing NDOs with placebo, reporting stool frequency, stool consistency, fecal pH, or short-chain fatty acids (SCFAs). Data were pooled using random-effects meta-analysis. All effect estimates are reported as standardized mean differences (SMDs) with 95% confidence intervals (CIs). Subgroups were analyzed based on baseline constipation status and treatment duration. **Results**: We included 20 RCTs (1786 participants) evaluating seven NDO types. NDO supplementation significantly increased stool frequency overall, with larger effects in constipated individuals (SMD 0.99, 95% CI 0.58–1.28) than in non-constipated population (SMD 0.30, 95% CI 0.10–0.51). By duration, shorter interventions (≤3 weeks) yielded greater frequency gains (SMD 0.89, 95% CI 0.40–1.38) than longer ones (SMD 0.24, 95% CI 0.09–0.38). While the overall effect on stool consistency was non-significant, constipated patients (SMD 0.46, 95% CI 0.19–0.74) and short-term trials (SMD 0.20, 95% CI 0.03–0.37) showed modest improvements. NDOs also lowered fecal pH (SMD −1.02, 95% CI −1.25–−0.79). Data on SCFAs were inconclusive and based on very limited studies. **Conclusions**: NDOs modestly increase stool frequency and lower fecal pH, with greater effects in constipated individuals and short-term interventions. However, evidence certainty remains low due to heterogeneity and study limitations. Further studies are needed to establish clinical utility.

## 1. Introduction

Functional constipation is a prevalent gastrointestinal disorder that impairs quality of life and imposes a considerable socioeconomic burden. A recent systematic review estimated a pooled global prevalence of approximately 14% among adults when contemporary Rome criteria are applied [1]. Beyond frequency alone, chronic constipation translates into reduced work productivity and daily activity impairment of 30–40%, with attendant healthcare costs approaching $1500 per patient annually [2]. Although bulk forming fibers, osmotic or stimulant laxatives, and newer secretagogues offer symptomatic relief, many patients experience sub optimal responses, adverse effects, or require long term pharmacotherapy that clinicians and patients alike deem unsatisfactory [3]. Consequently, there remains an unmet need for safe, diet-based strategies that target upstream gastrointestinal physiology to alleviate constipation.

Non-digestible oligosaccharides (NDOs) are short-chain (degree of polymerization 3–10) water-soluble carbohydrates, primarily including galacto-oligosaccharides (GOS), fructo-oligosaccharides (FOS), and mannooligosaccharides, which escape enzymatic digestion in the upper gut and are fermented by select saccharolytic microbes in the colon [4]. In vitro and animal studies show that NDO fermentation elevates luminal short-chain fatty acid (SCFA) concentrations. These metabolites stimulate enterochromaffin-cell serotonin biosynthesis and peptide-YY release via FFAR2 signalling, thereby accelerating colonic transit [5,6]. Parallel mechanistic works highlight additional benefits of NDO intake, including a lowering of colonic pH, expansion of mucus-associated Bifidobacterium populations, and shifting of microbial tryptophan catabolism toward health-promoting indole derivatives [7,8]. These processes—namely, increased fermentation products (SCFAs, and lactic acid) and osmotic load—are expected to increase fecal water content and bulk, resulting in higher stool frequency and softer consistency.

Despite these mechanistic insights, existing narrative reviews are often broad (“dietary fiber” or “prebiotics”), lack quantitative synthesis, and seldom dissect individual NDO species, doses, or target populations. Evidence from randomized controlled trials (RCTs) over the past decade is promising but heterogeneous. Placebo-controlled studies in adults with functional constipation show that GOS 5–11 g day^−1^ or inulin-type FOS 10 g day^−1^ can raise stool frequency by approximately one bowel movement per week and shift stool consistency towards Bristol Scale types 3–4 [9]. Similar magnitude effects have been reported for xylo-oligosaccharides (XOS) in otherwise healthy adults with low baseline frequency [10]. However, other trials in pediatric or elderly cohorts failed to detect significant improvements, possibly owing to short intervention periods, low NDO purity, or the use of maltodextrin rather than true non-fermentable placebos. A recent meta-analysis concentrated on FOS alone pooled 14 RCTs and confirmed significant gains in stool frequency yet could not resolve the high between-study heterogeneity [11]. The field therefore lacks an up-to-date, species-level and outcome-specific synthesis that simultaneously evaluates stool frequency and stool form while integrating gut-microbial and metabolic data.

We therefore undertook a systematic review and meta-analysis that quantifies the effects of seven NDOs on stool frequency and consistency across diverse populations, integrates concomitant microbial and fermentation data, and dissects heterogeneity by oligosaccharide type, treatment duration, and participant profile to generate clinically actionable guidance for NDO-based constipation management.

## 2. Methods

### 2.1. Literature Search Strategy

This systematic review and meta-analysis were conducted in accordance with the PRISMA 2020 guidelines [12]. The review protocol was registered in the International Prospective Register of Systematic Reviews (PROSPERO; Registration ID: CRD420251157704). We performed a comprehensive literature search using Ovid MEDLINE, Ovid Embase, and Web of Science, covering studies published in approximately the last 15 years (from 1 May 2010 to 1 May 2025). Search strings combined constipation-related terms with Medical Subject Headings (MeSH/Emtree) and free-text keywords for individual non-digestible oligosaccharides, e.g., “galacto-oligosaccharide*”, “fructo-oligosaccharide*”, “xylo-oligosaccharide*”, “mannan-oligosaccharide*”, “stachyose”, and “oligofructose”. A validated randomized trial filter was applied. Full strategies are provided in Table S1. Records were limited to human studies published in English. Reference lists of all included papers and relevant reviews were hand-searched to capture additional studies.

After removing duplicates, two reviewers (HC and JR) independently screened all titles and abstracts to identify potentially eligible studies, followed by full-text assessment of those articles. Any discrepancies in study selection were resolved by consultation with a third reviewer (RZ). To minimize the risk of missing relevant studies, the reference lists of all included articles and relevant reviews were also hand-searched. Study selection was guided by predefined inclusion criteria based on the PICOS framework (Population, Intervention, Comparator, Outcome, Study design) defined as follows: (1) participants (P): human subjects (including healthy individuals or patients with constipation); (2) interventions (I): nondigestible oligosaccharides (e.g., fructooligosaccharides, galactooligosaccharides, etc.); (3) comparators (C): other interventions not involving nondigestible oligosaccharides (for example, placebo or usual care); (4) outcomes (O): bowel movement frequency, stool consistency, stool pH, and short-chain fatty acid levels; and (5) study design (S): RCTs.

### 2.2. Inclusion and Exclusion Criteria

The inclusion criteria included the following: (1) use of a nondigestible oligosaccharide as the intervention; (2) use of a control intervention that did not include nondigestible oligosaccharides (e.g., placebo or other treatment); (3) a randomized controlled trial study design; and (4) participants who were healthy individuals or patients with constipation. The exclusion criteria included the following: (1) studies with incomplete or unavailable outcome data; (2) non-human or preclinical studies (animal or cell experiments), study protocols, or case reports; and (3) publications that were reviews, expert opinions or commentaries, conference abstracts, or letters.

### 2.3. Data Extraction and Risk of Bias Assessment

Data extraction was performed in accordance with the Cochrane Handbook for Systematic Reviews of Interventions. Two reviewers (HC and JR) independently extracted key data from each included study using a standardized data collection form, and any discrepancies were resolved by discussion. The following information was collected for each study: (1) study characteristics: first author’s name, publication year, country, sample size, study design, and type of control group; (2) participant characteristics: population type (healthy or constipated), mean age, and sex distribution of participants; (3) details of the intervention group: the specific nondigestible oligosaccharide used, its dosage, and the duration of the intervention; (4) details of the control group: description of the control or comparison intervention (e.g., placebo or alternative treatment) and its dosage/duration; and (5) outcome measures: definitions and values for outcomes of interest, including defecation frequency, stool form/consistency (e.g., as measured by a stool scale), fecal pH, and concentrations of short-chain fatty acids.

Two reviewers (HC and JR) also independently assessed the risk of bias for each included RCT using the Cochrane Risk of Bias 2 (RoB 2) tool recommended by the Cochrane Collaboration. The assessment covered the following domains: the randomization process, deviations from intended interventions, missing outcome data, outcome measurement, selection of reported results, and overall risk of bias. For each domain, trials were judged as having “low risk”, “some concerns”, or “high risk” of bias. Any disagreements in bias assessment were resolved through discussion or with input from another reviewer as needed. In addition, the overall certainty of the evidence for each outcome was appraised using the GRADE approach (Grading of Recommendations Assessment, Development and Evaluation), classifying the evidence quality into levels such as high, moderate, low, or very low based on factors like risk of bias, consistency of results, precision, and publication bias.

### 2.4. Data Synthesis and Analysis

All statistical analyses were carried out using Stata version 17.0. For continuous outcomes, we conducted meta-analyses using the post-intervention means and standard deviations from each study. Because the included studies used heterogeneous instruments and scales to measure the same indicator (e.g., stool frequency reported as events per week or per day and stool consistency reported as Bristol scores, Likert-type scales, or proportions of hard/soft/formed stools), we calculated pooled effects as standardized mean differences (SMDs) with 95% confidence intervals (CIs) to render the results comparable across studies. All outcomes are reported as SMDs with 95% CIs in this meta-analysis. The direction of the SMD was interpreted as follows: positive SMDs favor NDOs for stool frequency and consistency, whereas negative SMDs favor NDOs for fecal pH (reflecting greater acidification).

Between-study heterogeneity was assessed using Cochran’s Q (*p* < 0.10 indicating heterogeneity) and *I*^2^. When *I*^2^ > 50% or Q *p* < 0.10, we applied a random-effects model (DerSimonian–Laird); otherwise, a fixed-effect (inverse-variance) model was used. Sensitivity analyses included a leave-one-out approach and Galbraith plots to identify potentially influential/outlying studies (standardized residuals beyond ±1.96 flagged for scrutiny). Potential small-study effects/publication bias were explored by visual inspection of funnel plots (when ≥10 studies were available). Prespecified subgroup analyses evaluated effects by constipation status (constipated vs. non-constipated). In addition, a post hoc subgroup analysis of intervention duration (≤3 weeks vs. >3 weeks) was performed to further explore potential sources of heterogeneity.

## 3. Results

### 3.1. Study Selection and Characteristics

**Figure 1:**
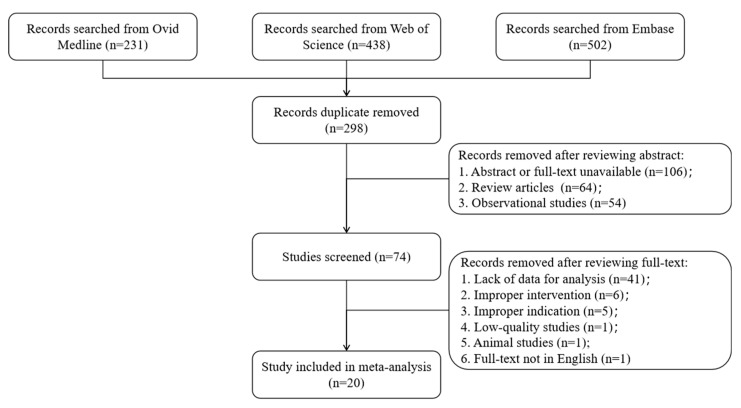
PRISMA Flowchart of literature search.

A total of 1171 records were identified through database searches, and 753 records remained after removing duplicates and were screened; ultimately 20 RCTs met the inclusion criteria and were included in the analysis (Figure 1). The 20 trials encompassed populations ranging from chronically constipated individuals to generally healthy infants, children, and adults. Seven trials were conducted in patients with functional or chronic constipation, whereas the remaining 13 trials enrolled healthy or non-constipated participants (including some with minor gastrointestinal complaints). All included studies evaluated a non-digestible oligosaccharide intervention (including GOS, deshipu stachyose granules (DSG), short-chain fructo-oligosaccharides/oligofructose (scFOS), inulin-type fructans, mannooligosaccharides (MOS), xylo-oligosaccharides (XOS), and partially hydrolyzed guar gum (PHGG)) versus a placebo. Daily NDO doses ranged from ~2.5 g to 30 g, and intervention durations ranged from 3 weeks to 12 months (typically 3–8 weeks in trials with adult participants). Study-level characteristics of the included RCTs are summarized in Table 1.

### 3.2. Risk of Bias

**Figure 2:**
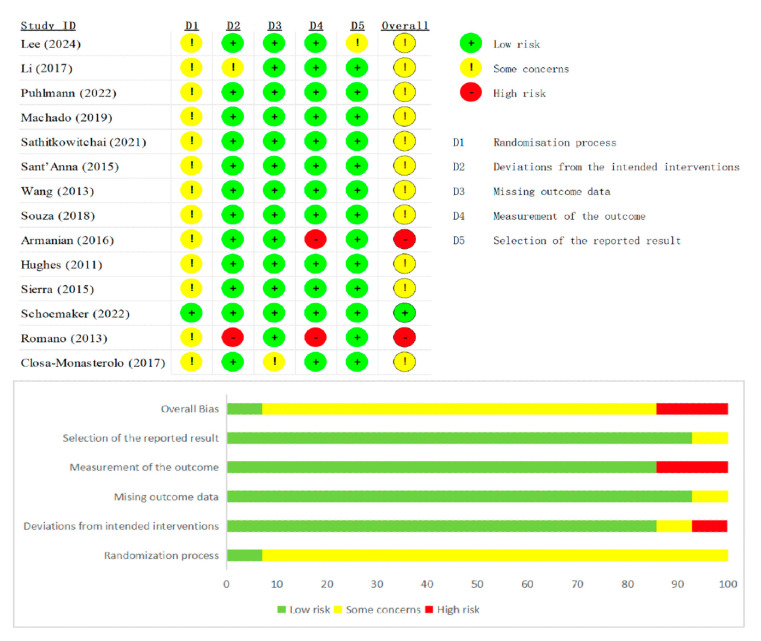
Summary plot of Cochrane Collaboration’s risk of bias tool 2 for all parallel RCT [9,13,14,16,18,19,20,21,23,24,25,27,29,31].

**Figure 3:**
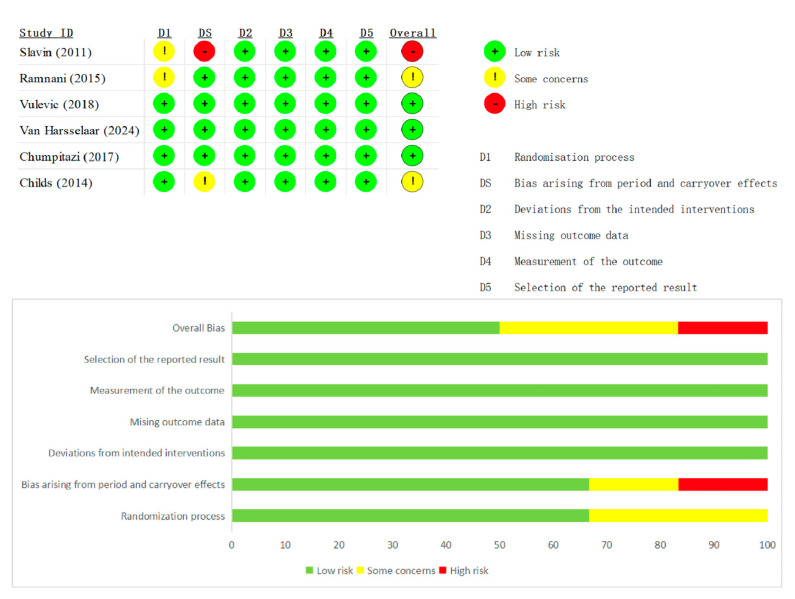
Summary plot of Cochrane Collaboration’s risk of bias tool 2 for all crossover RCT [15,17,22,26,28,30].

Risk-of-bias appraisal with the Cochrane RoB 2 tool (list as Figure 2 and Figure 3 below) showed generally sound methodology. Overall, 12 of 20 RCTs (60%) were judged low risk, 6 (30%) had some concerns, and 2 (10%) were high risk. In the 13 parallel trials, deficiencies were confined mainly to the randomization domain (≈70% “some concerns”), whereas blinding, attrition, and selective-reporting domains were largely low risk. In the seven crossover trials, risk profiles were similar except for carryover bias: five studies lacked an adequate wash-out and were therefore downgraded, accounting for most high-risk ratings. Across both designs, missing-data and outcome-measurement domains were seldom problematic, and only isolated studies exhibited serious selective-reporting bias. Sensitivity analyses that excluded the two high-risk trials left pooled estimates for stool frequency and consistency virtually unchanged, confirming that the main findings are robust to methodological limitations.

### 3.3. Stool Frequency

**Figure 4:**
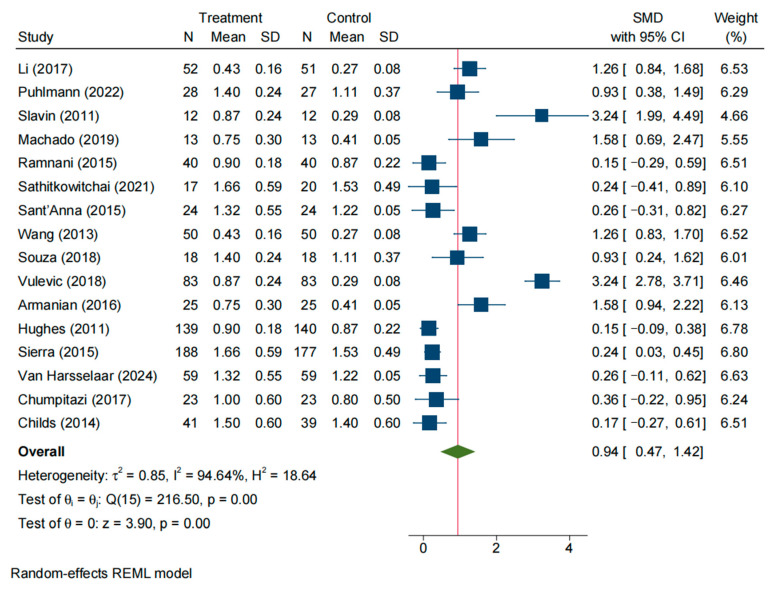
Forest plot of all studies reporting SMD and 95% CI for the effects of NDOs on stool frequency [13,14,15,16,17,18,19,20,21,22,23,24,25,26,28,30].

A total of 16 studies reported the frequency of defecation in the experimental and control groups. The random-effects model indicated substantial statistical heterogeneity among these studies (SMD = 0.19, 95% CI: −0.47–1.42; *I*^2^ = 94.64%, *p* < 0.05) (Figure 4). Sensitivity analysis (leave-one-out) (Figure S2A) and examination of the Galbraith plot (Figure S3A) and funnel plot (Figure S3B) identified Slavin (2011) [15] and seven other studies as outliers contributing to the heterogeneity. After excluding these studies, eight studies remained, and a fixed-effect model was applied. Heterogeneity was greatly reduced (*I*^2^ = 21.03%, *p =* 0.26), and the pooled effect size was 0.35 (95% CI: 0.17–0.52, *p* < 0.05). Translating this SMD, NDO supplementation increased bowel-movement frequency by approximately 1.59 events per week. This result indicates that the stool (bowel movement) frequency in the experimental group was significantly higher than that in the control group (Figure S1A).

#### 3.3.1. Effects on Stool Frequency in Constipated Individuals

Five studies reported defecation frequency in patients with constipation for both intervention and control groups. The random-effects model showed significant heterogeneity among these studies (SMD = 0.84, 95% CI: 0.41–1.28; *I*^2^ = 70.84%, *p* = 0.01). Sensitivity analysis and Galbraith plot suggested that Sant’Anna (2015) [19] was an outlier contributing considerable heterogeneity; after removing this study, four studies remained in the analysis. Using a random-effects model on these remaining studies, heterogeneity decreased but was still moderate (*I*^2^ = 60.97%, *p* = 0.06), and the pooled effect size was 0.99 (95% CI: 0.58–1.28, *p* < 0.05). This indicates that constipated patients receiving NDOs had a significantly higher defecation frequency than those receiving control, with a large effect size (approximately one extra bowel movement per week) (Figure S7A).

#### 3.3.2. Effects on Stool Frequency in Non-Constipated Individuals

Eleven studies reported defecation frequency in non-constipated individuals for intervention and control groups. The meta-analysis using a random-effects model revealed significant heterogeneity (SMD = 1.02, 95% CI: 0.33–1.70; *I*^2^ = 96.66%, *p* < 0.05). Sensitivity analysis and Galbraith plot identified Slavin (2011) [15] and five other studies as outliers contributing to the high heterogeneity; excluding these studies left five studies for analysis. A fixed-effect model on the remaining studies showed much lower heterogeneity (*I*^2^ = 31.63%, *p* = 0.21), and the combined effect size was 0.30 (95% CI: 0.10–0.51, *p* < 0.05). This finding suggests that non-constipated individuals in the intervention group also had a significantly higher frequency of bowel movements compared to the control group (Figure S7B).

#### 3.3.3. Effects on Stool Frequency by Intervention Duration (Post Hoc Analysis)

A subgroup analysis, performed post hoc in response to a reviewer’s suggestion, examined the effect of intervention duration (≤3 weeks vs. >3 weeks) and revealed differential effects. After excluding outliers, trials of shorter duration (≤3 weeks, *n* = 5) showed a larger increase in stool-movement frequency with NDO supplementation (SMD = 0.89, 95% CI: 0.40–1.38, *I*^2^ = 73.28%, *p* < 0.05; Figure S8A). In contrast, while still statistically significant, the effect size in longer-duration trials (>3 weeks, *n* = 4) was markedly smaller (SMD = 0.24, 95% CI: 0.09–0.38, *I*^2^ = 32.10%, *p* < 0.05; Figure S8B). Collectively, these results indicate that the efficacy of NDOs in increasing stool frequency is more pronounced in shorter intervention periods.

### 3.4. Stool Consistency

**Figure 5:**
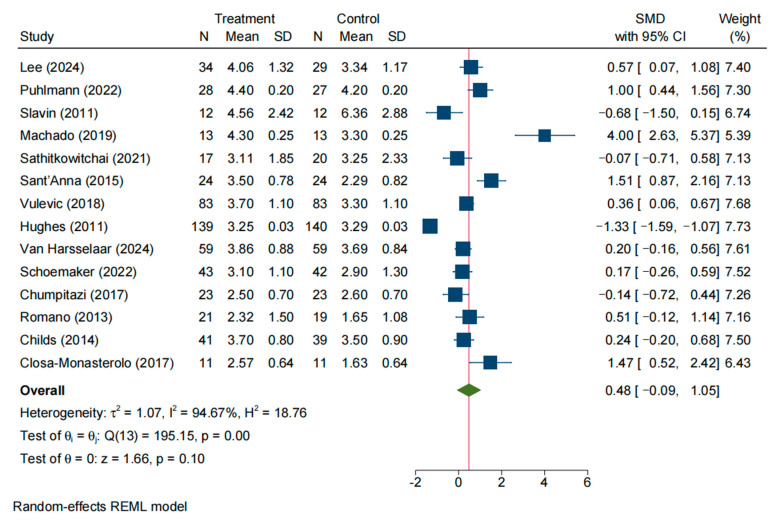
Forrest plot of all studies reporting SMD and 95% CI for the effects of NDOs on stool consistency [9,14,15,16,18,19,22,24,26,27,28,29,30,31].

A total of 14 studies evaluated stool consistency (e.g., stool form) in the intervention and control groups. The initial random-effects meta-analysis indicated significant heterogeneity across studies (SMD = 0.48, 95% CI: −0.09–1.05; *I*^2^ = 94.67%, *p* < 0.05) (Figure 5). Sensitivity analysis (Figure S2B) along with Galbraith (Figure S5A) and funnel plots (Figure S5B) suggested that Machado (2019) [16] and six other studies were outliers contributing to the heterogeneity. After removing these studies, seven studies remained; a fixed-effect model showed minimal heterogeneity (*I*^2^ = 9.58%, *p* = 0.36). The pooled effect size was SMD = 0.12 (95% CI: −0.07–0.32, *p* = 0.21), indicating no significant difference in stool consistency between the intervention and control groups (Figure S1B).

#### 3.4.1. Effects on Stool Consistency in Constipated Individuals

Six studies focused on stool consistency in constipated patients. The random-effects model showed considerable heterogeneity among these studies (SMD = 0.63, 95% CI: −0.11–1.15; *I*^2^ = 78.31%, *p* < 0.05). Sensitivity analysis and Galbraith plot indicated that Sant’Anna (2015) [19] and one other study were outliers contributing to heterogeneity; excluding these two studies left four studies in the analysis. Using a fixed-effect model on the remaining studies, heterogeneity was moderate but not statistically significant (*I*^2^ = 52.84%, *p* = 0.10). The pooled effect size was SMD = 0.46 (95% CI: 0.19–0.74, *p* < 0.05), suggesting that stool consistency in constipated patients was significantly improved in the intervention group compared to the control group (Figure S9A).

#### 3.4.2. Effects on Stool Consistency in Non-Constipated Individuals

Seven studies reported stool consistency outcomes in non-constipated individuals. The random-effects model showed no significant pooled effect and considerable heterogeneity (SMD = 0.39, 95% CI: −0.60–1.38; *I*^2^ = 97.47%, *p* < 0.05). Sensitivity analysis and the Galbraith plot identified Puhlmann (2022) [14] and two other studies as outliers contributing to the heterogeneity; after removing these studies, four studies remained. A fixed-effect model on these remaining studies indicated low heterogeneity (*I*^2^ = 30.24%, *p* = 0.23). The pooled effect size was SMD = 0.09 (95% CI: −0.15–0.34, *p* = 0.44), indicating no significant difference in stool consistency between the intervention and control groups among non-constipated participants (Figure S9B).

#### 3.4.3. Effects on Stool Consistency by Intervention Duration (Post Hoc Analysis)

The post hoc analysis also indicated that duration moderated the effect on stool consistency. After excluding outliers, a meta-analysis of shorter-duration trials (≤3 weeks, *n* = 6) showed negligible heterogeneity and a small but significant improvement in stool consistency (SMD = 0.20, 95% CI: 0.03–0.37, *I*^2^ = 0%, *p* < 0.05; Figure S10A). In contrast, the analysis of longer-duration trials (>3 weeks, *n* = 6) was non-significant and highly heterogeneous (SMD = 1.05, 95% CI: −0.31–2.40, *I*^2^ = 96.96%, *p* = 0.13; Figure S10B). Taken together, the evidence points to a small, early improvement in stool consistency that has not been demonstrated beyond 3 weeks.

### 3.5. Faecal pH

Three studies reported stool pH levels for the intervention and control groups. Since no significant heterogeneity was detected (*I*^2^ = 0%, *p* = 0.53), a fixed-effect model was used for the meta-analysis. The combined effect size was −1.02 (95% CI: −1.25–−0.79, *p* < 0.05), indicating that the stool pH in the intervention group was significantly lower than that in the control group (Figure S11). In other words, participants in the intervention group had more acidic stool compared to controls, and this difference was statistically significant.

### 3.6. Total Short-Chain Fatty Acids

Only two studies reported outcomes for total SCFAs in stool. The results showed a high level of heterogeneity between these two studies (*I*^2^ = 93.22%, *p* < 0.05), so a random-effects model was applied. The pooled effect size was 1.19 (95% CI: −1.47–3.86, *p* = 0.38), suggesting no significant difference in total SCFA levels between the intervention and control groups (Figure S12). These findings are considered inconclusive due to the extremely limited number of studies.

## 4. Discussion

Our meta-analysis found that NDOs provide modest benefits for constipation-related outcomes, with effects moderated by baseline status and intervention duration. NDOs significantly increased stool frequency by approximately one bowel movement per week overall, but this effect was substantially larger in constipated patients and in shorter-term trials (≤3 weeks). For stool consistency, a modest softening was observed specifically in constipated subgroups and within the short-term period. Concurrently, NDOs lowered fecal pH, indicative of increased colonic fermentation. It is important to highlight, however, that our pooled analysis revealed no conclusive evidence for a rise in total fecal SCFA levels. This SCFA finding is considered preliminary and inconclusive, being based on only two studies and the inherent limitations of fecal SCFA measurement.

Our results are biologically plausible given the known actions of NDOs. These fermentable carbohydrates (including GOS, FOS, XOS, and MOS, etc.) resist digestion in the upper gut and reach the colon intact. There, they are selectively fermented by beneficial colonic microbiota such as *Bifidobacterium* and *Lactobacillus* [32]. This fermentation is expected to produce SCFAs, which are understood to have pro-motility and osmotic effects in the colon. For example, SCFAs stimulate intestinal peristalsis by triggering serotonin (5-HT) release from enterochromaffin cells, thereby activating neural reflexes that accelerate colonic transit [33]. Meanwhile, the fermentative breakdown of fiber increases colonic osmotic load and draws water into the lumen, resulting in greater fecal water content, bulkier volume, and softer stools. It is plausible that these mechanisms contribute to the improved bowel function observed in our meta-analysis, although our data do not directly confirm an increase in fecal SCFAs. In parallel, the accumulation of organic acids (SCFAs and lactic acid) from NDO fermentation lowers luminal pH (acidifies the stool), which is an expected consequence of enhanced saccharolytic activity [34]. Together, these processes directly explain the observed increases in stool frequency and the softer stool consistency with NDO supplementation.

Previous evidence syntheses are consistent with laxative efficacy of non-digestible oligosaccharides. Three recent high-quality systematic reviews, focusing respectively on scFOS and GOS, each reported mean increases of approximately 0.7–1.2 bowel movements week^−1^ and parallel softening of stool form in both constipated and free-living cohorts [35,36]. A review pooling 25 prebiotic trials further confirmed a class-wide ability of NDOs, regardless of chain length or botanical origin, to accelerate colonic transit and lower luminal pH, thereby supporting the generalizability of our findings [37]. Regulatory appraisal echoes this evidence: the European Food Safety Authority concluded that ≥12 g day^−1^ of native chicory inulin reliably “increases stool frequency”, establishing a cause-and-effect relationship between inulin-type fructans and normal defecation [38]. This concordance is reassuring but does not obviate the limitations discussed below; accordingly, we characterize average effects as modest and context-dependent rather than uniformly “significant” across all settings.

The interpretation of our findings must be tempered by several important limitations pertaining to the strength and generalizability of the available evidence. First, the overall certainty of evidence is low to moderate, primarily due to unexplained heterogeneity across studies. High *I*^2^ values, particularly for stool frequency outcomes, reflect clinical and methodological diversity in participant populations, NDO types and dosages, and intervention durations. Second, the body of evidence is constrained by the scale and design of primary studies. Most trials were characterized by small sample sizes and short-term interventions, limiting robust assessment of long-term efficacy and safety. Furthermore, key patient subgroups, including pediatric and elderly populations, were notably underrepresented. Third, the interpretation of several outcomes is limited by methodological inconsistencies and a sparse evidence base. For instance, biomarkers like fecal pH and SCFAs were reported in only a subset of trials, and even among those, laboratory methods were not standardized. Furthermore, our meta-analysis for SCFAs was based on merely two highly heterogeneous studies, rendering the finding inconclusive. Finally, while sensitivity analyses supported the robustness of primary outcomes and most domains exhibited low risk of bias, certain methodological concerns remain. These include inadequate description of randomization in some trials and potential carryover effects in crossover designs. Although funnel plots did not provide conclusive evidence of publication bias, the possibility cannot be ruled out, particularly for outcomes with limited numbers of small studies.

From a clinical perspective, NDO supplementation should be positioned as a dietary adjunct rather than a replacement for first-line pharmacologic therapies. The ability of NDOs to increase stool frequency and modestly soften stool consistency has tangible implications for patient management. For individuals suffering from infrequent, difficult defecation, adding a daily prebiotic supplement (such as an inulin, fructo-oligosaccharide, or galacto-oligosaccharide preparation) could help normalize bowel function and reduce reliance on stimulant laxatives. Unlike some pharmacologic laxatives, prebiotic fibers work by gently enhancing the body’s natural peristalsis and stool hydration, and they have a favorable safety profile. In the included trials, NDOs were generally well tolerated; while mild flatulence and bloating were relatively common, these effects were usually transient and rated as tolerable by participants, and no serious adverse events were attributed to NDOs at typical doses (often around 5–15 g per day). Pragmatically, gradual dose titration and tailoring NDO type to individual tolerance/response are advisable, and the observed pH-lowering with enrichment of beneficial taxa hints at broader gut-health benefits—hypothesis-generating signals that warrant confirmation in longer, standardized studies.

Four research priorities will add precision and clinical traction to the NDO field. First, dose-finding and safety trials of longer duration are needed. Most data cluster around 10–15 g day^−1^, yet it is unclear whether higher intakes offer extra benefit or adverse effect; multi-center RCTs with ≥6-month follow-up should define the therapeutic ceiling. Second, head-to-head efficacy studies should compare GOS, DSG, scFOS, inulin-type fructans, MOS, XOS, and PHGG, alone or in combination, to pinpoint the most potent or synergistic formulations. Third, precision targeting deserves attention: stratifying participants by baseline microbiota, transit phenotype, age, and comorbidity (e.g., diarrhea-predominant irritable bowel syndrome, paediatrics, and frailty) will clarify who benefits most and reduce heterogeneity. Finally, the potential synergistic use of NDOs with probiotic strains (synbiotics) is an area for exploration: combining prebiotics with specific probiotic bacteria might amplify beneficial effects on gut motility and stool characteristics. Addressing these gaps will translate NDO science into nuanced, patient-centered constipation care.

## 5. Conclusions

This systematic review suggests that NDOs may modestly increase bowel-movement frequency and lower fecal pH. Potential benefits were observed across NDO types and in both constipated and non-constipated populations, with more pronounced effects on stool frequency and stool consistency in short-term (≤3 weeks) trials. However, the certainty of evidence is low to moderate, limited by high heterogeneity, small studies, and short durations. No conclusive findings on SCFAs could be drawn due to limited data. While NDOs appear safe as a potential dietary adjunct, they are not a replacement for first-line therapies. Larger, well-designed, longer trials in diverse groups are needed to confirm durability, identify responders, and refine clinical recommendations.

## Figures and Tables

**Table 1:** Characteristics of included studies.

Author (Year)	Population	Study Type and Duration	Duration	Sample Size and Intervention/Control Group	Intervention		Outcome		
					Intervention Group	Control Group	Bowel Function (Frequency and Consistency)	Fecal Microbiota (Notable Changes)	Fecal Metabolites (pH and SCFAs)
Lee (2024) [9]	Adults with functional constipation	Double-blinded, parallel RCT	4 weeks	34/29	Galacto-oligosaccharides capsule 333.33 mg/capsule (plus maltodextrin excipient), 3 capsules twice daily	392 mg maltodextrin/capsule, 3 capsules twice daily	↑ Bowel movements: Int: +0.36 from baseline: 0.42 ± 0.20 → 0.78 ± 0.31/day (*p* < 0.05); Int: +0.15/day than Ctrl (*p* = 0.0480) ↑ Stool Consistency (BSS): 4.06 ± 1.32 (Int) vs. 3.34 ± 1.17 (Ctrl), *p* = 0.0283	Int vs. baseline: ↑ Bifidobacterium (*p* = 0.0047), ↑ Lactobacillus (*p* = 0.0182), Int vs. Ctrl: - Bifidobacterium (*p* > 0.05), - Lactobacillus (*p* > 0.0182)	pH (NS) SCFA (NR)
Li (2017) [13]	Adults with functional constipation	Open label, parallel RCT	30 days	50/50	Deshipu stachyose granules (DGS) 5 g/day	Maltodextrin 5 g/day	↑ Bowel movements: Int: +1.24 from baseline: 1.78 ± 0.51 → 3.02 ± 1.15/week (*p* < 0.05); Ctrl: 1.92 ± 0.60 → 1.86 ± 0.58/week (NS) 3.02 ± 1.15 (Int) vs. 2.11 ± 0.92 (Ctrl), *p* < 0.05 ↑ Stool Consistency (BSS): Int: 1.66 ± 0.48 → 1.23 ± 0.33, *p* < 0.05; Ctrl: 1.58 ± 0.44 → 1.37 ± 0.41 (NS) 1.23 ± 0.33 (Int) vs. 1.37 ± 0.41(Ctrl), *p* < 0.05	↑ Bifidobacterium (7.2 → 7.8 log10 CFU/g, *p* < 0.01) ↑ Lactobacillus (6.8 → 7.5 log10 CFU/g, *p* < 0.01) ↓ *Clostridium perfringens* (*p* < 0.05)	pH (NR) SCFA (NR)
Puhlmann (2022) [14]	Adults at risk for type 2 diabetes	Investigator-blinded, parallel RCT	3 weeks	28/27	Dried chicory root (70% inulin) 30 g/day	Maltodextrin 16 g/day	↑ Bowel movements: Int: 1.3 ± 0.1 → 1.9 ± 0.1/day, *p* = 0.004 Ctrl: 1.2 ± 0.1 → 1.3 ± 0.1/day, *p* = 0.582 (NS) ↑ Stool Consistency (BSS): Int: 3.3 ± 0.3 → 4.4 ± 0.2, *p* = 0.034 Ctrl: 4.1 ± 0.2 → 4.2 ± 0.2, *p* = 0.69 (NS)	↑ Bifidobacterium (4.09-fold, *p* < 0.001) ↑ Anaerostipes (3.24-fold, *p* < 0.001)	pH (NR) SCFA: ↑ Total SCFA (+13.02 ± 6.26 mmol/kg, *p* = 0.023); ↑ acetate (+9.25 mmol/kg, *p* = 0.022); ↑ butyrate (+1.75 mmol/kg, *p* = 0.052)
Slavin (2011) [15]	Healthy adult, males, aged 27–49 years	Double-blinded, crossover RCT	3 weeks	12/12	Chicory inulin 20 g/day (basal diet with the addition of 20 g of chicory inulin)	Low-fiber control diet	- Bowel movements (self-scored line): 9.80 ± 1.70 (Int) vs. 7.80 ± 2.59 (Ctrl), *p* = 0.14 - Stool Consistency (self-scored line): Int: 4.56 ± 2.42 (Int) vs. 6.36 ± 2.88 (Ctrl), *p* = 0.10	↑ Total anaerobes (*p* = 0.03) ↑ *Lactobacillus* spp. (*p* = 0.05) - *Bifidobacterium* spp. (*p* = 0.33) - *Clostridium* spp. (*p* = 0.05) - *Bacteroides* spp. (*p* = 0.05)	pH (NS) SCFA: ↑ Acetate : propionate (ratio), (*p* = 0.0005)
Machado (2019) [16]	Adults with overweight	Double-blinded, parallel RCT	6 weeks	13/13	Yacon flour 25 g/day (providing 0.1 g FOS/kg body weight/day)	Corn starch, dose equivalent to intervention	- Bowel movements: Constipation frequency Int: 1.92 ± 0.44 → 1.46 ± 0.17, *p* < 0.05 Ctrl: 1.38 ± 0.17 → 1.92 ± 0.36 (NS) Loose stools frequency Int: 1.69 ± 0.22 → 2.53 ± 0.43, *p* < 0.05 Ctrl: 1.69 ± 0.33 → 1.46 ± 0.30 (NS) Hard stools frequency Int: 2.61 ± 0.50 → 1.61 ± 0.25 (NS) Ctrl: 1.84 ± 0.21 → 1.77 ± 0.29 - Stool Consistency (BSS): Int: 3.92 ± 0.29 → 4.30 ± 0.25 (NS) Ctrl: 3.38 ± 0.27 → 3.30 ± 0.25 (NS)	NR	pH (NS): 7.16 ± 0.14 → 7.32 ± 0.11 (NS) SCFA (NR)
Ramnani (2015) [17]	Healthy adults	Double-blinded, crossover RCT	3 weeks	40/40	Agave fructans 5 g/day	Maltodextrin 5 g/day	- Bowel movements: Int: 1.2 ± 0.4 → 1.4 ± 0.5/day (NS) Ctrl: 1.1 ± 0.3 → 1.3 ± 0.4/day (NS) - Stool Consistency: Hard stools (%): Int: 6.9 ± 15.2 → 6.6 ± 11.6 (NS) Ctrl: 7.3 ± 11.4 → 7.9 ± 17.1 (NS) Formed stools (%): Int: 73.4 ± 22.4 → 55.0 ± 34.9 (NS) Ctrl: 74.1 ± 25.1 → 71.3 ± 27.0 (NS) Soft stools (%): Int: 20.1 ± 25.1 → 38.5 ± 36.4 (NS) Ctrl: 16.9 ± 23.2 → 20.5 ± 22.1 (NS)	↑ *Bifidobacterium* spp. (9.2 ± 0.4 → 9.6 ± 0.4, *p* < 0.001) ↑ Lactobacillus (7.3 ± 0.6 → 7.7 ± 0.8, *p* < 0.001)	pH (NR) SCFA - Acetate 0.7 ± 0.4 → 0.8 ± 0.7 (NS) - Propionate 0.3 ± 0.2 → 0.3 ± 0.3 (NS) - Butyrate 0.2 ± 0.1 → 0.2 ± 0.2 (NS) - Total SCFA 1.2 ± 1.3 → 1.1 ± 1.2 (NS)
Sathitkowitchai (2021) [18]	Healthy adults	Double-blinded, parallel RCT	3 weeks	20/17	Mannooligosaccharides 5 g/day	Maltodextrin, 10 g/day	- Bowel movements: Int (3 g CMH): 1.07 ± 0.51 → 1.02 ± 0.45/day (NS) Ctrl: 1.00 ± 0.48 → 1.11 ± 0.59/day (NS) ↑ Stool Consistency (% of total stools): Int: 2.51 ± 1.25 → 1.99 ± 0.84, *p* = 0.021 Ctrl: 2.50 ± 1.14 → 3.25 ± 2.33 (NS)	- Actinobacteria (%) (0.95 ± 1.98 → 1.47 ± 1.87, *p* = 0.108) - Bacteroidetes (%) (3.67 ± 8.86 → 6.43 ± 10.49, *p* = 0.328) - Firmicutes (%) (85.89 ± 8.75 → 88.54 ± 14.18, *p* = 0.424) - Proteobacteria (%) (1.99 ± 2.53 → 1.62 ± 5.85, *p* = 0.351) - Firmicutes: Bacteroidetes (ratio) (23.95 ± 61.92 → 13.92 ± 20.89, *p* = 0.301)	pH (NR) SCFA (NR)
Sant’Anna (2015) [19]	Constipated adults	Parallel, maltodextrin-controlled, randomization and blinding design was not reported	30 days	24/24	Fructooligosaccharides/inulin, 10 g/day	Maltodextrin, 25 g/day	↑ Bowel movements: Int: 2.04 ± 0.95 → 6.08 ± 1.69/week, *p* < 0.001 Ctrl: 1.92 ± 0.78 → 2.00 ± 0.59/week (NS) ↑ Stool Consistency Int: 2.29 ± 0.90 → 3.50 ± 0.78, *p* < 0.001 Ctrl: 2.33 ± 0.82 → 2.29 ± 0.78 (NS)	↑ Bifidobacterium (*p* < 0.05) ↓ Clostridium (*p* < 0.05) ↓ Enterobacteriaceae (*p* < 0.05) ↓ Lactobacillus (*p* < 0.05)	↓ pH Int: 6.98 ± 0.74 → 6.56 ± 0.53, *p* = 0.006 Ctrl: 7.15 ± 0.72 → 7.17 ± 0.67 (NS) SCFA - Acetate Int: 64.74 ± 24.59 → 61.83 ± 34.11 (NS) Ctrl: 63.00 ± 33.00 → 60.68 ± 29.10 (NS) - Butyrate Int: 25.25 ± 15.77 → 25.84 ± 15.29 (NS) Ctrl: 17.65 ± 4.15 → 18.33 ± 3.43 (NS) - Propionate Int: 27.67 ± 23.31 → 26.06 ± 18.68 (NS) Ctrl: 31.87 ± 19.80 → 31.77 ± 22.57 (NS) - Lactate Int: 4.50 ± 3.01 → 4.86 ± 3.05 (NS; ↑ vs. Ctrl, *p* = 0.02) Ctrl: 3.27 ± 0.96 → 3.05 ± 1.48 (NS)
Wang (2013) [20]	Constipated adults	Double-blinded, parallel RCT	10 days	50/50	Fructooligosaccharides capsules 1.26 g/day	Starch (dose equivalent to intervention)	↑ Bowel movements: Int: 2.54 ± 0.50 → 5.24 ± 2.11/week, *p* < 0.01 Ctrl: 2.74 ± 0.44 → 2.86 ± 0.35/week (NS) ↑ Stool Consistency (BSS): Int: 1.54 ± 0.61 → 0.56 ± 0.58, *p* < 0.01 Ctrl: 1.08 ± 0.27 → 1.00 ± 0.40 (NS)	NR	pH (NR) SCFA (NR)
Souza (2018) [21]	Infants with constipation	Double-blinded, parallel RCT	4 weeks	19/19	Fructooligosaccharides, 6–12 g/day (dose based on weight: 6.0–8.9 kg: 6 g/day; 9.0–11.9 kg: 9 g/day; ≥12 kg: 12 g/day)	Maltodextrin, same dosing regimen	- Bowel movements: Int: 6.27 ± 1.32 → 6.33 ± 1.28/week, *p* = 0.320 Ctrl: 5.66 ± 1.87 → 6.11 ± 1.53/week, *p* = 0.133 ↑ Stool Consistency (% of bowel movements): Int: 12.12 ± 15.91 → 73.38 ± 29.38, *p* = 0.035 Ctrl: 16.92 ± 15.07 → 55.38 ± 36.32 (NS)	↑ Bifidobacterium (log CFU/g, median) (6.39 (5.25–8.36) → 7.37 (5.86–8.43), *p* = 0.006) - Lactobacillus (log CFU/g, median) 6.27 (4.33–7.54) → 6.45 (4.83–7.61) (*p* = 0.095, NS)	pH (NR) SCFA (NR)
Vulevic (2018) [22]	Adults with bloating, abdominal pain or flatulence	Double-blinded, crossover RCT	2 weeks	83/83	Bimuno^®^-Galactooligosaccharides 2.75 g/day (1.37 g active GOS)	Maltodextrin, 2.75 g/day	- Bowel movements: Int: 11.9 ± 4.7 → 11.6 ± 4.1/week (NS) Ctrl: 10.6 ± 5.0 → 10.7 ± 3.4/week (NS) - Stool Consistency (BBS): Int: 3.7 ± 1.1 → 3.7 ± 1.1 (NS) Ctrl: 3.4 ± 1.1 → 3.3 ± 1.1 (NS)	NR	pH (NR) SCFA (NR)
Armanian (2016) [23]	Preterm neonates	Parallel RCT	1 week	25/25	Short-chain galacto-oligosaccharides/long-chain fructo-oligosaccharides, initial 0.5 g/kg/day, gradually increased to 1.5 g/kg/day	Distilled water, dose equivalent to intervention	↑ Bowel movements: Int: 1.9 ± 1.4 → 2.6 ± 1.0/day, *p* = 0.014 Ctrl: 1.6 ± 1.0 → 2.2 ± 0.9/day, *p* = 0.133 (NS) ↑ Stool Consistency: 2.4 ± 0.4 (Int) versus 1.9 ± 0.5 (Ctrl) /day, *p* = 0.003	NR	pH (NR) SCFA (NR)
Hughes (2011) [24]	Healthy university students	Double-blinded, parallel RCT	8 weeks	140/139	Galacto-oligosaccharides, 2.5 g/day and 5.0 g/day	Bakers’ sugar (sucrose), dose equivalent to intervention	- Bowel movements: 1.50 ± 0.03 (0 g GOS) vs. 1.47 ± 0.03 (2.5 g GOS) vs. 1.51 ± 0.03 (5.0 g GOS) (NS) Stool Consistency (BBS): - 2 g GOS: 3.29 ± 0.03 ↑ 2.5 g GOS: 3.42 ± 0.03 (*p* < 0.05 vs. 0 g) - 5.0 g GOS: 3.25 ± 0.03	NR	pH (NR) SCFA (NR)
Sierra (2015) [25]	Healthy infants	Double-blinded, parallel RCT	4 months	188/177	Galacto-oligosaccharides, infant formula 0.44 g/dL, follow-on formula 0.50 g/dL	Standard infant and follow-on formulas without GOS	↑ Bowel movements: 3 mo: 1.45 ± 0.97 (Int) vs. 1.26 ± 0.83 (Ctrl), *p* < 0.05 4 mo: 1.50 ± 0.99 (Int) vs. 1.26 ± 0.94 (Ctrl), *p* < 0.05 6–12 mo: NS ↑ Stool Consistency (% cases) Hard stools (3 mo): 1.9% (Int) vs. 6.4% (Ctrl) *p* < 0.05 Loose stools (3 mo): 17.7% (Int) vs. 13.5% (Ctrl), *p* < 0.05 Lumpy stools (3 mo): 35.7% (Int) vs. 21.9% (Ctrl), *p* < 0.05	↑ *Bifidobacterium* spp. (log_10_ CFU/g, 4 mo) 8.65 ± 1.31 (Int) vs. 8.02 ± 1.66 (Ctrl), *p* = 0.010 - *Lactobacillus* spp. 7.11 ± 0.89 (Int) vs. 6.87 ± 0.61 (Ctrl), *p* = 0.151 (NS) - *Bacteroides* spp. 7.12 ± 2.04 (Int) vs. 6.83 ± 2.17 (Ctrl), *p* = 0.187 (NS) - Clostridium difficile positive rate 45.2% (Int) vs. 63.3% (Ctrl), *p* = 0.037 - *Bifidobacterium breve* 6.37 ± 1.57 (Int) vs. 4.93 ± 1.44 (Ctrl), *p* = 0.055	↓ pH (4 mo): 6.57 ± 0.82 (Int) vs. 7.09 ± 0.69 (Ctrl), *p* = 0.019 SCFA (4 mo) - Total SCFAs (µmol/g) 93.36 ± 40.70 (Int) vs. 98.91 ± 39.56 (Ctrl), *p* = 0.567 (NS) - Acetate (% of SCFAs) 84.77 ± 8.52 (Int) vs. 75.72 ± 9.76 (Ctrl), *p* = 0.005 - Propionate (% of SCFAs) 11.53 ± 6.62 (Int) vs. 15.27 ± 6.30 (Ctrl), *p* = 0.015 - Butyrate (% of SCFAs) 2.29 ± 2.29 (Int) vs. 5.59 ± 4.06 (Ctrl), *p* = 0.040
Van Harsselaar (2024) [26]	Healthy male adults	Double-blinded, crossover RCT	2 weeks	32/32	Oligofructose, 2.5 g/day	Maltodextrin, 2.5 g/day	↑ Bowel movements: Int: 7.91 ± 3.02 → 9.27 ± 3.88/week, *p* = 0.014 Ctrl 8.67 ± 3.36 → 8.53 ± 3.34/week, *p* = 0.713 (NS) 9.27 ± 3.88 (Int) vs. 8.53 ± 3.34 (Ctrl), *p* = 0.0204 - Stool Consistency (BSS): Int: 3.73 ± 0.91 → 3.86 ± 0.88, *p* = 0.158 (NS) Ctrl: 3.71 ± 0.86 → 3.69 ± 0.84, *p* = 0.782 (NS) 3.86 ± 0.88 (Int) vs. 3.69 ± 0.84 (Ctrl), *p* = 0.1684 (NS)	↑ Bifidobacterium (log_10_ copies/g) (7.61 ± 0.58 → 7.88 ± 0.54, *p* = 0.016)	pH (NR) SCFA (NR)
Schoemaker (2022) [27]	Constipated adults	Double-blinded, parallel RCT	3 weeks	45/43	Galacto-oligosaccharides, 11 g/day or 5.5 g/day	Maltodextrin, 15.1 g/day	↑ Bowel movements: 11 g GOS: 2.9 ± 1.2 → 3.9 ± 1.8, *p* = 0.027 (subgroup ≤3/week) 5.5 g GOS: 3.4 ± 1.9 → 3.6 ± 2.0, *p*= 0.36 (NS) Ctrl: 3.0 ± 1.2 → 3.2 ± 1.6, *p*= 0.41 (NS) - Stool Consistency 11 g GOS: 2.7 ± 1.2 → 3.1 ± 1.1, *p* = 0.37 (NS) 5.5 g GOS: 2.8 ± 1.3 → 2.8 ± 1.3, *p* = 0.62 (NS) Ctrl: 2.7 ± 1.3 → 2.9 ± 1.3, *p* = 0.41 (NS)	↑ Bifidobacterium: 11 g GOS: 10.9% → 23.9%, *p* < 0.001 5.5 g GOS: 11.6% → 19.1%, *p* = 0.16 (NS) ↑ Anaerostipes hadrus 11 g GOS: adjusted *p* = 0.03 5.5 g GOS: adjusted *p* = 0.57 (NS)	pH NR - SCFA (*p* > 0.05)
Chumpitazi (2017) [28]	Children with irritable bowel syndrome	Double-blinded, crossover RCT	72 h	23/23	Inulin-type fructan, 0.5 g/kg/day (maximum 19 g/day)	Maltodextrin, 0.5 g/kg/day (maximum 19 g/day)	- Bowel movements: Int: 0.9 ± 0.5 → 1.0 ± 0.6/day, *p* = 0.08 (NS) Ctrl: 0.9 ± 0.4 → 0.8 ± 0.5/day, *p* = 0.42 (NS) 1.0 ± 0.6 (Int) vs. 0.8 ± 0.5 (Ctrl), *p* = 0.08 (NS) - Stool Consistency: Int: 2.6 ± 0.7 → 2.5 ± 0.7, *p* = 0.56 (NS) Ctrl: 2.5 ± 0.7 → 2.6 ± 0.7, *p* = 0.60 (NS) 2.5 ± 0.7 (Int) vs. 2.6 ± 0.7 (Ctrl), *p* = 0.56 (NS)	NR	pH (NR) SCFA (NR)
Romano (2013) [29]	Children with chronic abdominal pain or irritable bowel syndrome	Parallel RCT	4 weeks	30/30	Partially hydrolyzed guar gum, 5 g/day in fruit juice	Unsupplemented fruit juice	Bowel movements (NR); ↑ Stool Consistency: IBS-C 1.00 ± 1.02 → 2.02 ± 1.50 (Int), *p* < 0.05 1.16 ± 0.89 → 1.76 ± 1.04 (Ctrl), *p* = 0.09 (NS) IBS-D 5.02 ± 0.63 → 4.01 ± 0.16 (Int), *p* < 0.05 5.54 ± 0.32 → 4.86 ± 0.96 (Ctrl), *p* = 0.08 (NS) Normalization (Bristol type 3–4): 40% (Int) vs. 13.3% (Ctrl), *p* = 0.025	NR	pH (NR) SCFA (NR)
Childs (2014) [30]	Healthy adults	Double-blinded, crossover RCT	21 days	41/39	Xylo-oligosaccharides, 8 g/day	Maltodextrin, 8 g/day	↑ Bowel movements: Int: 1.4 ± 0.6 → 1.5 ± 0.6/day, *p* = 0.005 Ctrl: 1.4 ± 0.6 → 1.4 ± 0.6/day (NS) 1.5 ± 0.6 (Int) vs. 1.4 ± 0.6 (Ctrl), *p* = 0.005 - Stool Consistency: Int: 3.5 ± 0.9 → 3.7 ± 0.8, *p* = 0.10 (NS) Ctrl: 3.5 ± 0.9 → 3.5 ± 0.9 (NS) 3.7 ± 0.8 (Int) vs. 3.5 ± 0.9 (Ctrl), *p* = 0.097 (NS)	↑ *Bifidobacterium* spp. (log_10_ cells/g dry) (9.8 ± 0.7 → +0.3 ± 0.5 (XOS), *p* = 0.008)	pH NR - SCFA - Acetate: −2.5 ± 25.8 (Int) vs. +3.6 ± 26.9 (Ctrl) - Butyrate: −0.8 ± 11.4 (Int) vs. +2.1 ± 13.1 (Ctrl)
Closa-Monasterolo (2017) [31]	Functional constipated children	Double-blinded, parallel RCT	6 weeks	8/9	Inulin-type fructans, 4 g/day	Maltodextrin, 4 g/day	- Bowel movement: Int: 3.33 ± 1.74 → 4.63 ± 1.14/week (NS) Ctrl: 5.73 ± 3.03 → 5.94 ± 3.24/week (NS) ↑ Stool Consistency: Int: 2.19 ± 0.55 → 2.60 ± 0.58, *p* = 0.040 Ctrl: 1.74 ± 0.54 → 1.63 ± 0.64, *p* = 0.487 (NS)	NR	pH (NR) SCFA (NR)

RCT = randomized controlled study. Int = Intervention group. Ctrl = Control group. ↑ = significant increase (*p* < 0.05) vs. control; ↓ = significant decrease (*p* < 0.05) vs. control; ‘-’ or NS = no significant change; NR = not reported; Bristol = Bristol Stool Scale score; SCFA = short-chain fatty acids. mo = month.

## Data Availability

No new data were created or analyzed in this study. Data sharing is not applicable to this article.

## Supplementary Materials

The following supporting information can be downloaded at https://www.mdpi.com/article/10.3390/nu17203246/s1. Table S1. Search strategy. Figure S1. Forrest plot reporting SMD and 95% CI for the effects of NDOs on (A) stool frequency, and (B) stool consistency, after excluding biased studies. Figure S2. Sensitivity analysis was performed by removing each study in turn to determine the impact of each study on the overall effect size of NDOs on (A) stool frequency, and (B) stool consistency. Figure S3. Galbraith plot (A) and funnel plot (B) representing publication bias of all studies reporting effect size of NDOs on stool frequency. Figure S4. Galbraith plot (A) and funnel plot (B) representing publication bias of studies reporting effect size of NDOs on stool frequency, after excluding biased studies. Figure S5. Galbraith plot (A) and funnel plot (B) representing publication bias of all studies reporting effect size of NDOs on stool consistency. Figure S6. Galbraith plot (A) and funnel plot (B) representing publication bias of studies reporting effect size of NDOs on stool consistency, after excluding biased studies. Figure S7. Forrest plot reporting SMD and 95%CI for the effects of NDOs on stool frequency in (A) constipated patients, and (B) healthy individuals, after excluding biased studies. Figure S8. Forrest plot of all studies reporting SMD and 95%CI for the effects of NDOs on stool frequency by intervention duration: (A) ≤3 weeks; (B) >3 weeks, after excluding biased studies. Figure S9. Forrest plot reporting SMD and 95%CI for the effects of NDOs on stool consistency in (A) constipated patients, and (B) healthy individuals, after excluding biased studies. Figure S10. Forrest plot of all studies reporting SMD and 95%CI for the effects of NDOs on stool consistency by intervention duration: (A) ≤3 weeks; (B) >3 weeks, after excluding biased studies. Figure S11. Forrest plot reporting SMD and 95%CI for the effects of NDOs on fecal pH. Figure S12. Forrest plot reporting SMD and 95%CI for the effects of NDOs on fecal total SCFA.

## Abbreviations

The following abbreviations are used in this manuscript:

NDO(s)	Non-digestible oligosaccharide(s)
RCT(s)	Randomized controlled trial(s)
GOS	Galacto-oligosaccharides
FOS	Fructo-oligosaccharides
scFOS	Short-chain fructo-oligosaccharides (oligofructose)
XOS	Xylo-oligosaccharides
MOS	Mannooligosaccharides
PHGG	Partially hydrolyzed guar gum
DSG	Deshipu stachyose granules
SCFA(s)	Short-chain fatty acid(s)
FFAR2	Free fatty acid receptor 2
