# Supplementary material for: Non-Digestible Oligosaccharides and Constipation: A Systematic Review and Meta-Analysis of Randomized Trials on Stool Frequency, Stool Consistency, and Fermentation Biomarkers
Source: Nutrients. 2025 Oct 16;17(20):3246. doi: 10.3390/nu17203246 (PMC12567251; doi:10.3390/nu17203246)
Supplement: Supplementary file 1 [file nutrients-17-03246-s001.zip › nutrients-3900183-supplementary.pdf]

nutrients

MDPI

Systematic Review

Non-Digestible Oligosaccharides and Constipation: A Systematic Review and Meta-Analysis of Randomized Trials on Stool Frequency, Stool Consistency, and Fermentation Biomarkers

Huiyu Chen [1,†], Jiale Ren [2,†], Langrun Wang [1], Wenyi Zhang [3], Sufang Duan [3], Jie Guo [1], Qingshan Chen [3], Ran Wang [1], Jian He [3], Jingjing He [4,*] and Ruixin Zhu [1,*]

[1] Key Laboratory of Precision Nutrition and Food Quality, Department of Nutrition and Health, China Agricultural University, Beijing 100083, China
[2] School of Public Health, Capital Medical University, Beijing 100069, China
[3] National Technology Innovation Center for Dairy, Hohhot 010100, China; hejian@yili.com
[4] Research Center for Probiotics, China Agricultural University, Beijing 100083, China
[*] Correspondence: hejingjing89@cau.edu.cn (J.H.); zhuruixin07@126.com (R.Z.)
[†] These authors contributed equally to this work.

Supplementary Material

1. Supplementary Figures and Tables

1.1. Supplementary Tables

Table S1. Search strategy.

Ovid MEDLINE		
#	Query	Results from May 2025
1	exp Constipation/ or constipation.mp. or colonic inertia.mp. or dyschezia.mp. or opioid-induced constipation.mp. or chronic constipation.mp. or functional constipation.mp. or severe constipation.mp. or bowel habit*.mp. or bowel movement*.mp. or stool frequency.mp. or stool consistency.mp. or defecation.mp. or gut transit.mp. or gastrointestinal transit.mp.	60,925
2	(fructooligosaccharide* or FOS or kestose or nystose or fructofuranosylnystose).ab,ti.	35,602
3	(isomaltooligosaccharide* or IMO or isomaltose or panose or isomaltotetraose or isomaltopentaose).ab,ti.	1,742
4	exp Xylans/ or xylooligosaccharide*.ab,ti. or XOS.ab,ti. or xylobiose.ab,ti. or xylotriose.ab,ti. or xylotetraose.ab,ti.	5,300
5	exp Mannans/ or mannan-oligosaccharide*.ab,ti. or MOS.ab,ti. or mannobiose.ab,ti. or mannotriose.ab,ti.	17,934

6	exp Galactans/ or galactooligosaccharide*.ab,ti. or GOS.ab,ti. or lactulose.ab,ti. or lactosucrose.ab,ti. or galactobiose.ab,ti.	23,734
7	(soybean oligosaccharide* or SOS or raffinose or stachyose).ab,ti.	13,087
8	exp Chitosan/ or exp Chitin/ or chitosan oligosaccharide*.ab,ti. or COS.ab,ti. or chitooligosaccharide*.ab,ti.	60,296
9	(nondigestible oligosaccharide* or non-digestible oligosaccharide* or prebiotic oligosaccharide*).ab,ti.	486
10	or/2-9	154,315
11	1 and 10	1,302
12	(randomized controlled trial or controlled clinical trial).pt. or randomized.ab. or randomly.ab. or placebo.ab. or trial.ab. or groups.ab.	4,050,481
13	animals/ not humans/	5,286,024
14	12 not 13	3,517,664
15	11 and 14	575
16	limit 15 to (english language and humans and last 15 years)	231
Ovid Embase		
#	Query	Results from May 2025
1	exp constipation/ or constipation.mp. or colonic inertia.mp. or dyschezia.mp. or opioid-induced constipation.mp. or chronic constipation.mp. or functional constipation.mp. or severe constipation.mp. or bowel habit*.mp. or bowel movement*.mp. or stool frequency.mp. or stool consistency.mp. or defecation.mp. or gut transit.mp. or gastrointestinal transit.mp.	173,086
2	exp fructooligosaccharide/ or exp fructan/ or fructooligosaccharide*.ab,ti. or FOS.ab,ti. or kestose.ab,ti. or nystose.ab,ti. or fructofuranosylnystose.ab,ti.	46,106
3	(isomaltooligosaccharide* or IMO or isomaltose or panose or isomaltotetraose or isomaltopentaose).ab,ti.	2,145
4	exp xylooligosaccharide/ or exp hemicellulose/ or xylooligosaccharide*.ab,ti. or XOS.ab,ti. or xylobiose.ab,ti. or xylotriose.ab,ti. or xylotetraose.ab,ti.	10,819
5	(mannan-oligosaccharide* or MOS or mannobiose or mannotriose).ab,ti. or exp mannan/	31,631
6	exp galactooligosaccharide/ or galactooligosaccharide*.ab,ti. or GOS.ab,ti. or lactulose.ab,ti. or lactosucrose.ab,ti. or galactobiose.ab,ti.	15,719
7	(soybean oligosaccharide* or SOS or raffinose or stachyose).ab,ti.	15,605
8	exp chitosan/ or exp chitin/ or chitosan oligosaccharide*.ab,ti. or COS.ab,ti. or chitooligosaccharide*.ab,ti.	86,193

9	(nondigestible oligosaccharide* or non-digestible oligosaccharide* or prebiotic oligosaccharide*).ab,ti.	634
10	or/2-9	205,345
11	1 and 10	2,633
12	limit 11 to (human and english language and (clinical trial or randomized controlled trial or controlled clinical trial or multicenter study or phase 1 clinical trial or phase 2 clinical trial or phase 3 clinical trial or phase 4 clinical trial) and last 15 years)	502
Web of Science		
#	Query	Results from May 2025
1	TS=(constipation OR "colonic inertia" OR dyschezia OR "opioid-induced constipation" OR "chronic constipation" OR "functional constipation" OR "severe constipation" OR "bowel habit*" OR "bowel movement*" OR "stool frequency" OR "stool consistency" OR defecation OR "gut transit" OR "gastrointestinal transit")	56951
2	TS=(fructooligosaccharide* OR FOS OR kestose OR nystose OR fructofuranosylnystose OR isomaltooligosaccharide* OR IMO OR isomaltose OR panose OR isomaltotetraose OR isomaltopentaose OR xylooligosaccharide* OR XOS OR xylobiose OR xylotriose OR xylotetraose OR mannan-oligosaccharide* OR MOS OR mannobiose OR mannotriose OR galactooligosaccharide* OR GOS OR lactulose OR lactosucrose OR galactobiose OR "soybean oligosaccharide*" OR SOS OR raffinose OR stachyose OR "chitosan oligosaccharide*" OR COS OR chitooligosaccharide* OR nondigestible oligosaccharide* OR "non-digestible oligosaccharide*" OR "prebiotic oligosaccharide*")	179702
3	TS=("randomized controlled trial*" OR RCT* OR random* OR placebo* OR "clinical trial*" OR "controlled trial*" OR groups OR trial)	9844590
4	#3 AND #2 AND #1 Timespan: 2010-05-01 to 2025-05-01	450
5	(#4) AND LA==("ENGLISH")	438

1.2. Supplementary Figures

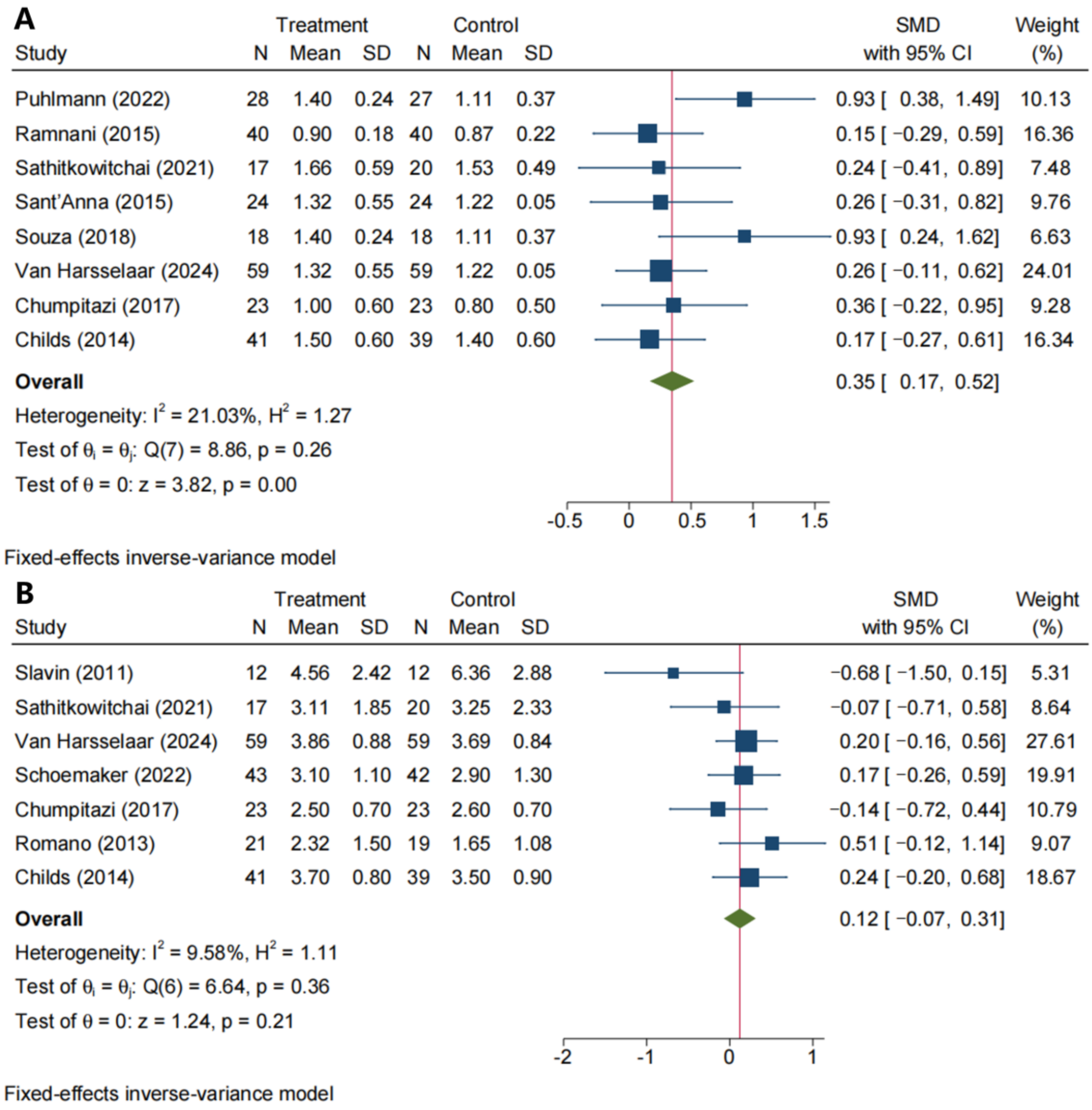

Figure S1. Forrest plot reporting SMD and 95%CI for the effects of NDOs on (**A**) stool frequency, and (**B**) stool consistency, after excluding biased studies.

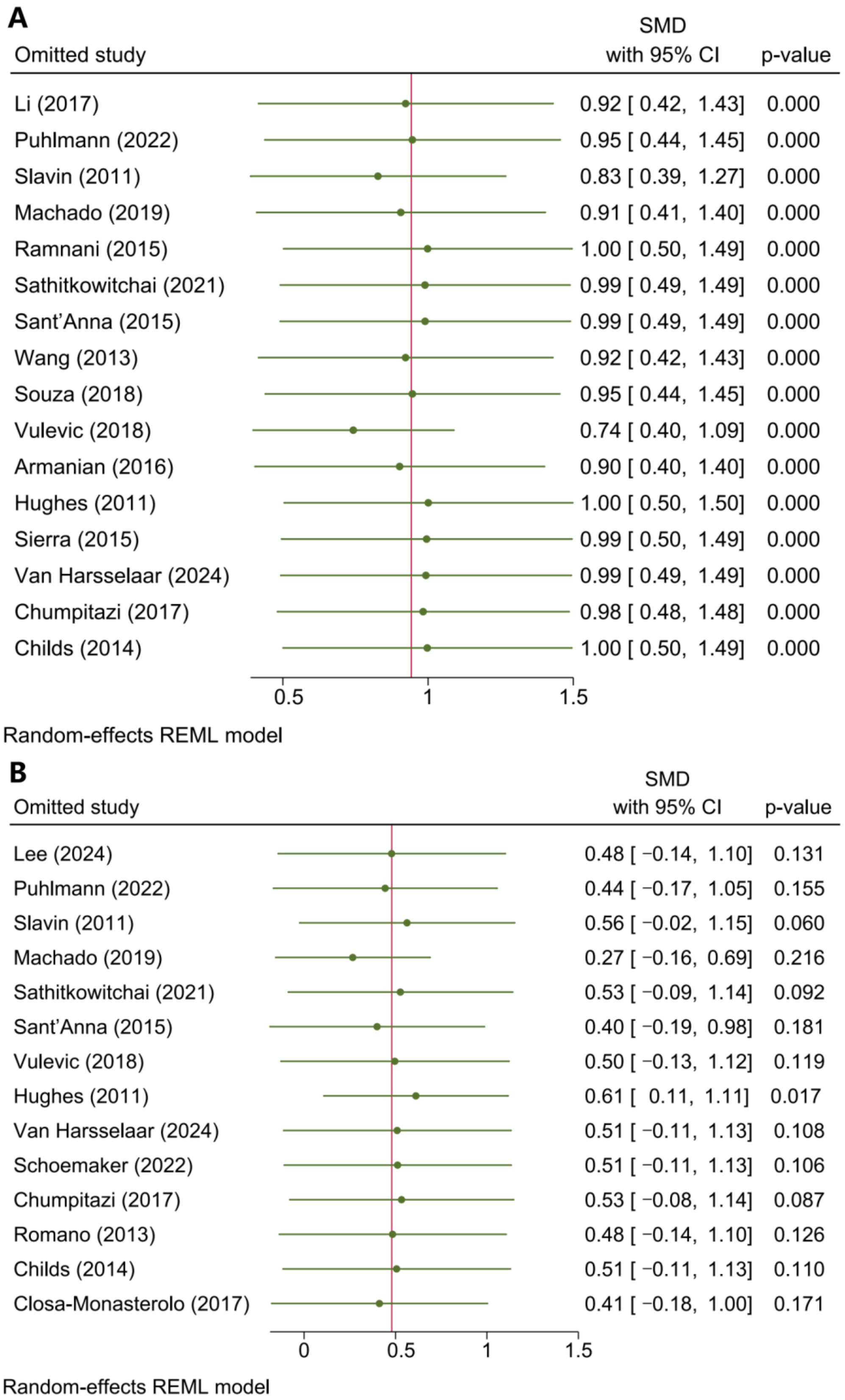

Figure S2. Sensitivity analysis was performed by removing each study in turn to determine the impact of each study on the overall effect size of NDOs on (**A**) stool frequency, and (**B**) stool consistency.

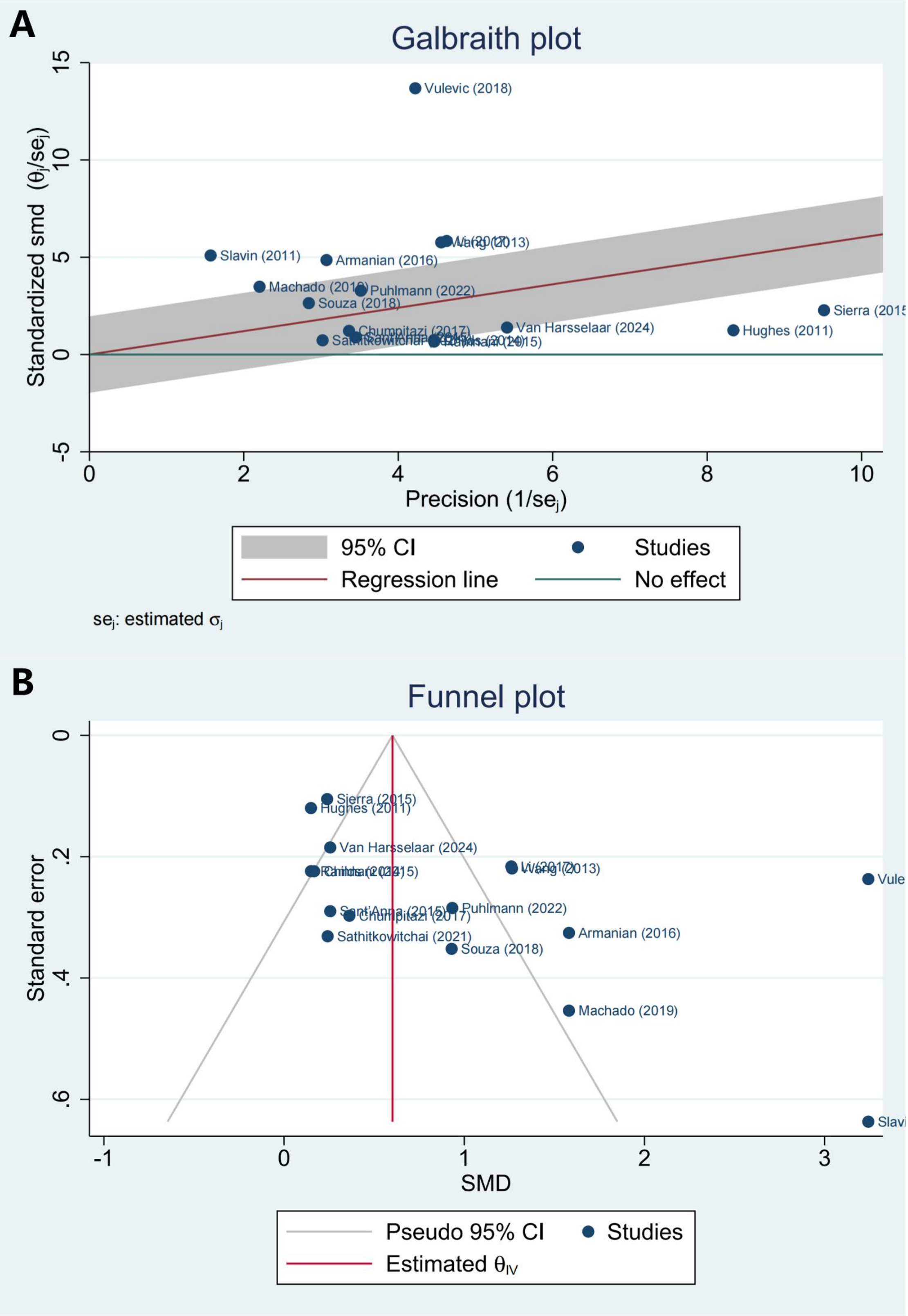

Figure S3. Galbraith plot (**A**) and funnel plot (**B**) representing publication bias of all studies reporting effect size of NDOs on stool frequency.

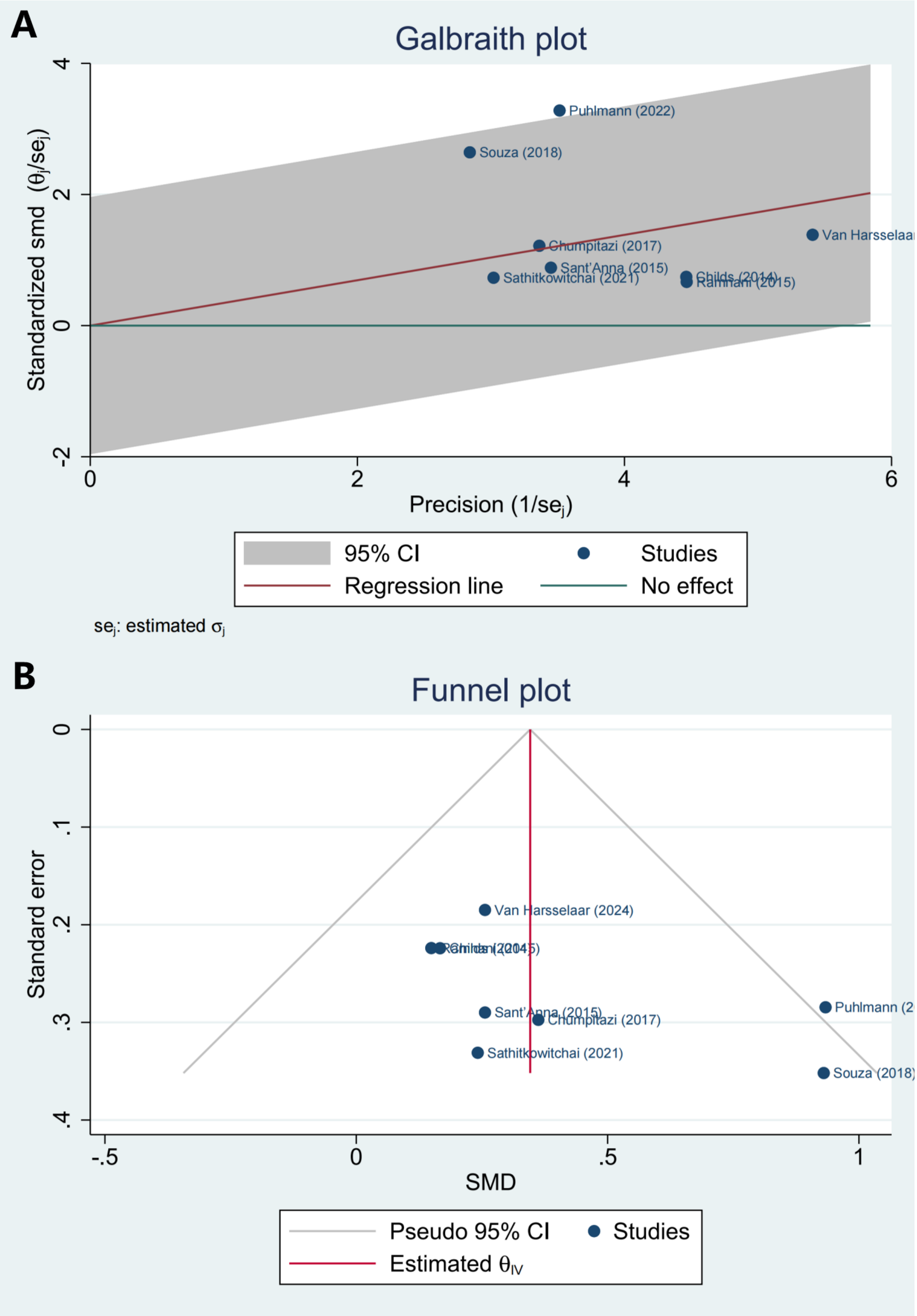

Figure S4. Galbraith plot (**A**) and funnel plot (**B**) representing publication bias of studies reporting effect size of NDOs on stool frequency, after excluding biased studies.

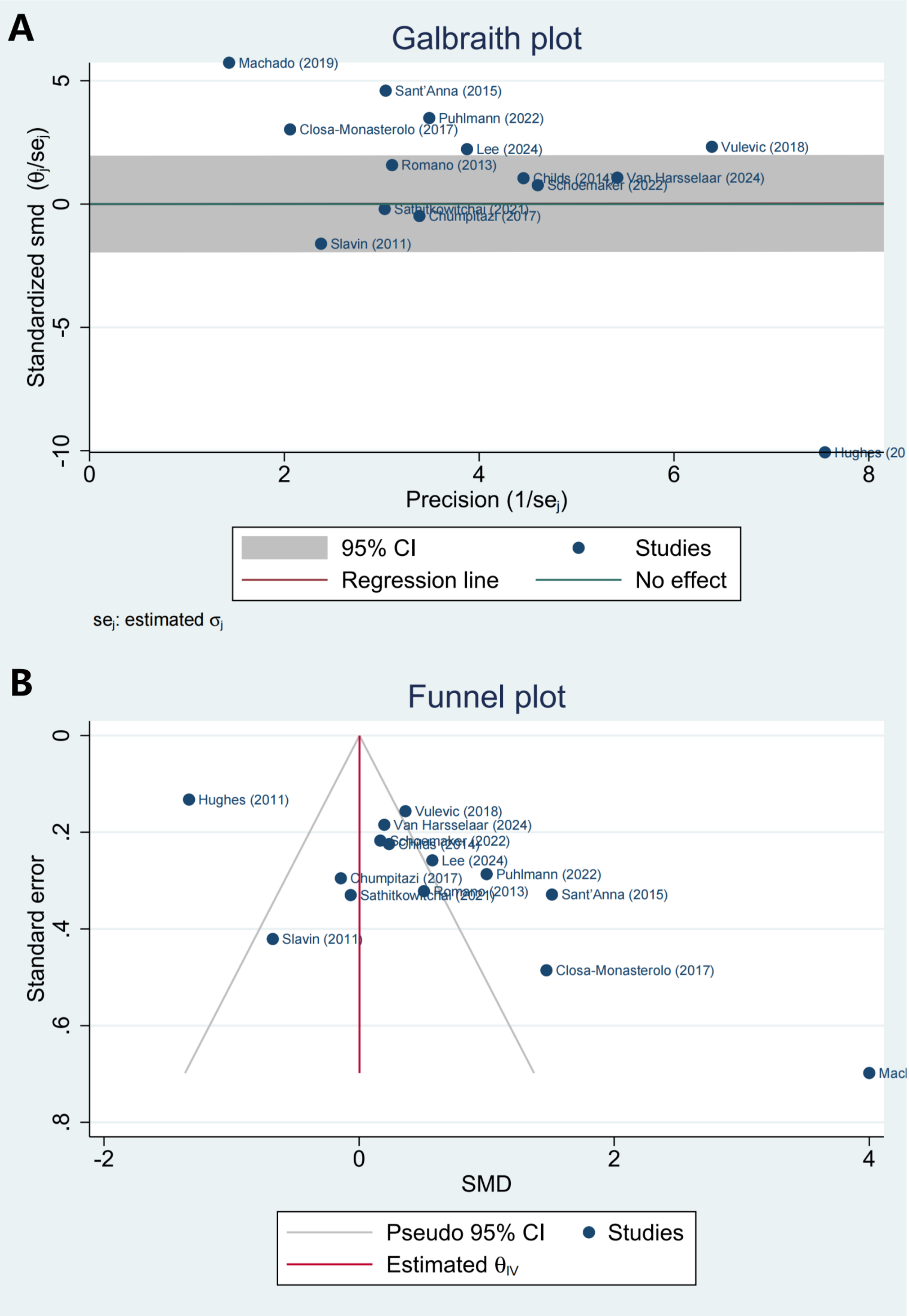

Figure S5. Galbraith plot (**A**) and funnel plot (**B**) representing publication bias of all studies reporting effect size of NDOs on stool consistency.

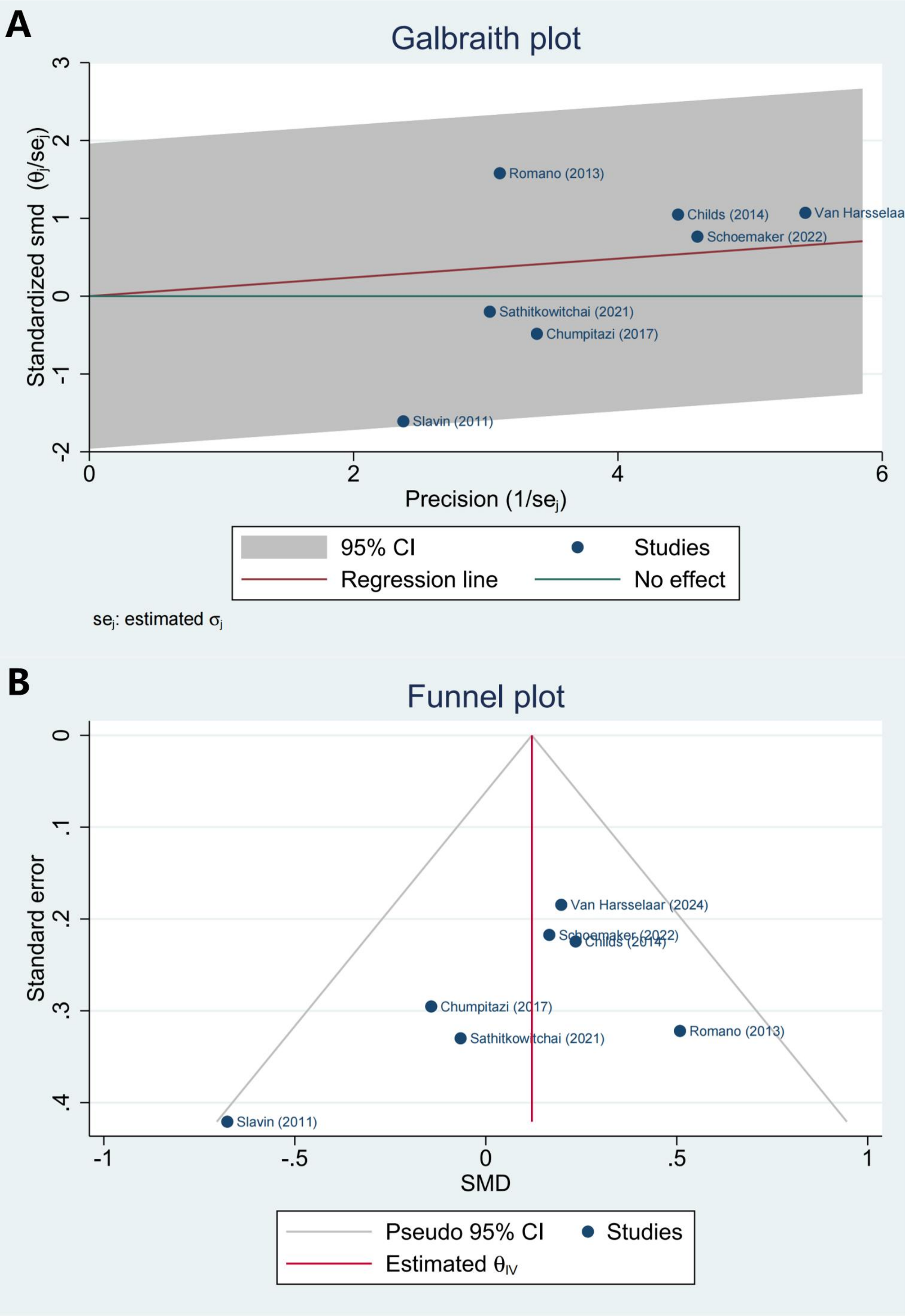

Figure S6. Galbraith plot (**A**) and funnel plot (**B**) representing publication bias of studies reporting effect size of NDOs on stool consistency, after excluding biased studies.

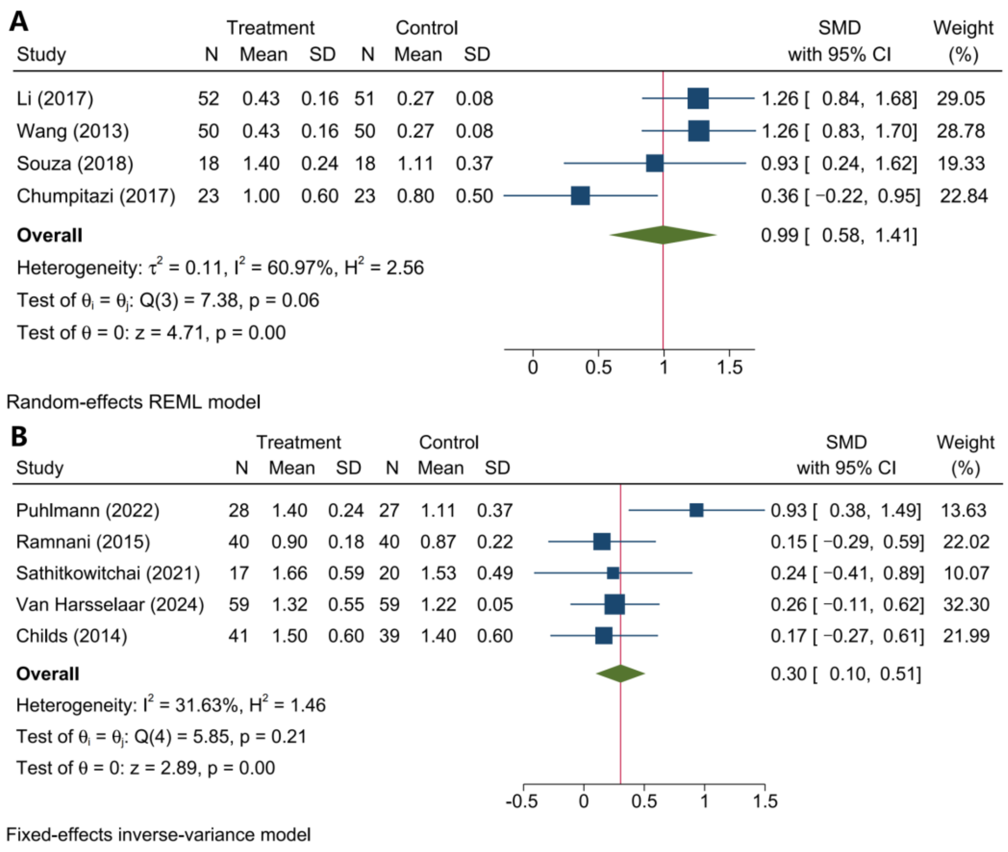

Figure S7. Forrest plot reporting SMD and 95%CI for the effects of NDOs on stool frequency in (**A**) constipated patients, and (**B**) healthy individuals, after excluding biased studies.

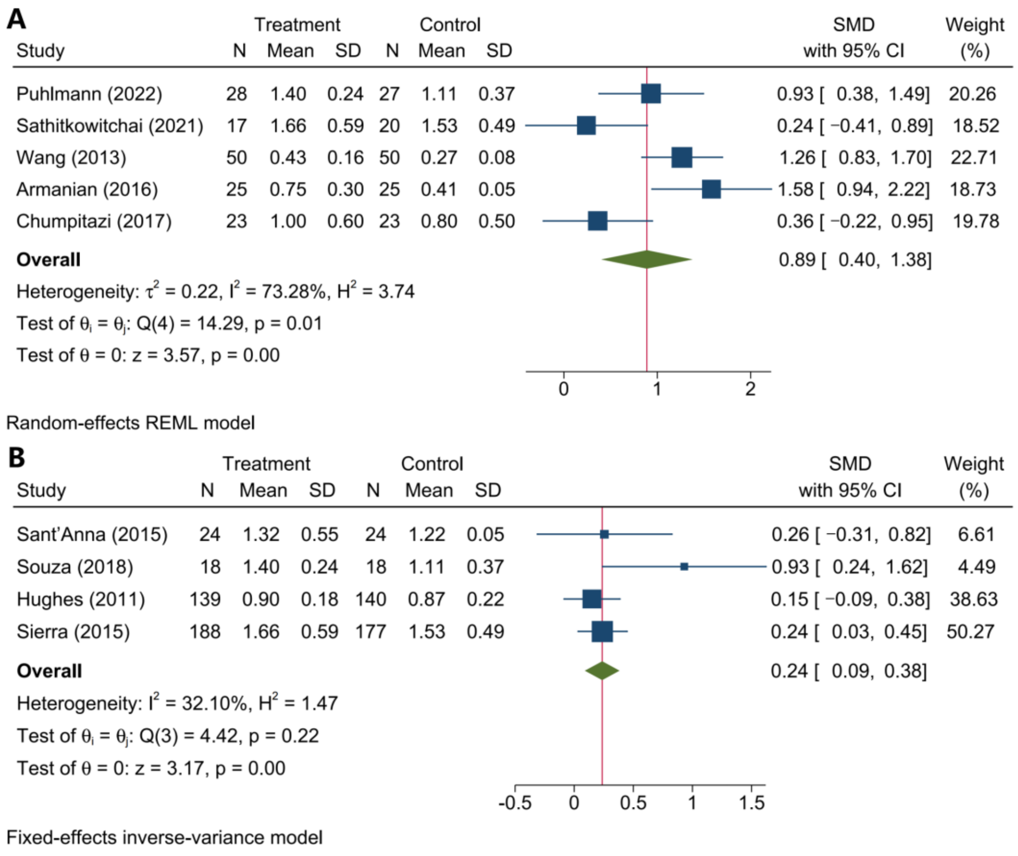

Figure S8. Forrest plot of all studies reporting SMD and 95%CI for the effects of NDOs on stool frequency by intervention duration: (**A**) ≤3 weeks; (**B**) >3 weeks, after excluding biased studies.

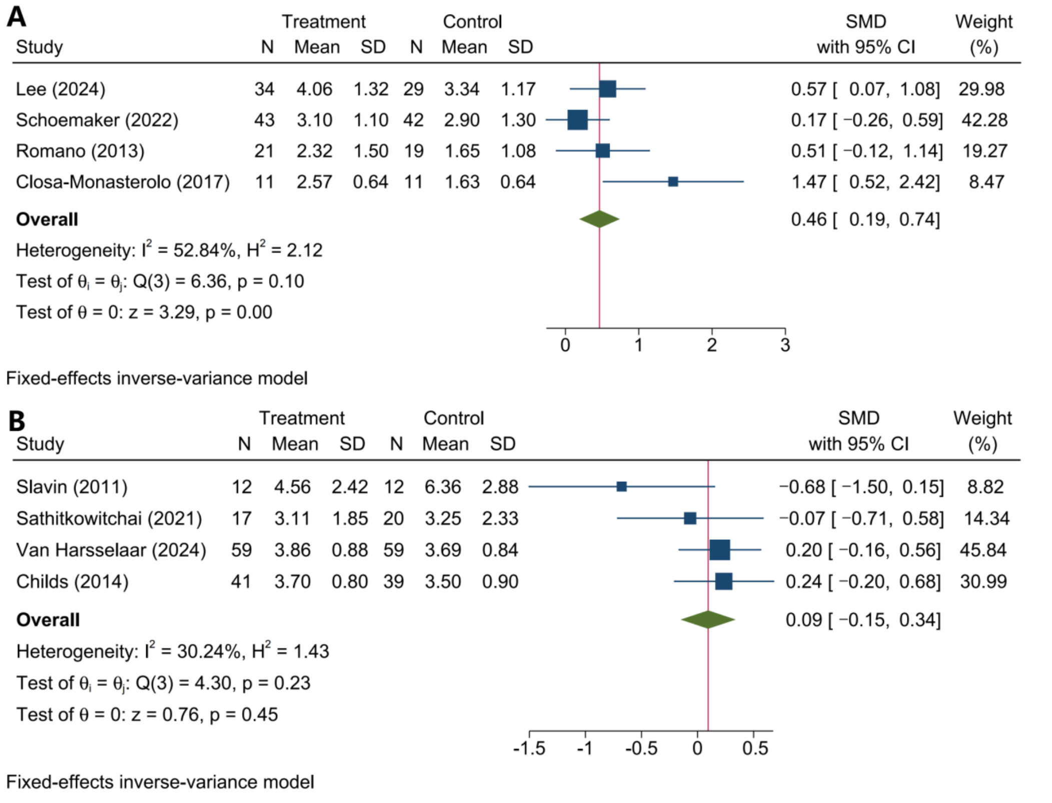

Figure S9. Forrest plot reporting SMD and 95%CI for the effects of NDOs on stool consistency in (**A**) constipated patients, and (**B**) healthy individuals, after excluding biased studies.

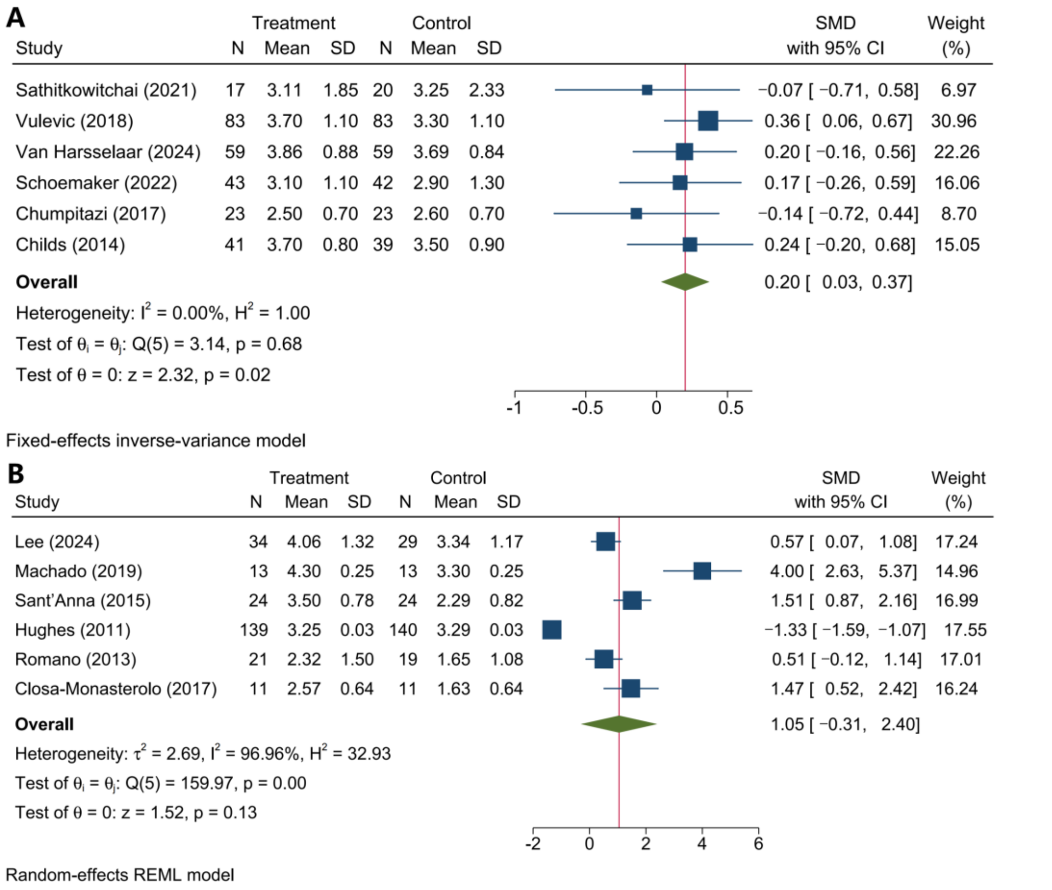

Figure S10. Forrest plot of all studies reporting SMD and 95%CI for the effects of NDOs on stool consistency by intervention duration: (**A**) ≤3 weeks; (**B**) >3 weeks, after excluding biased studies.

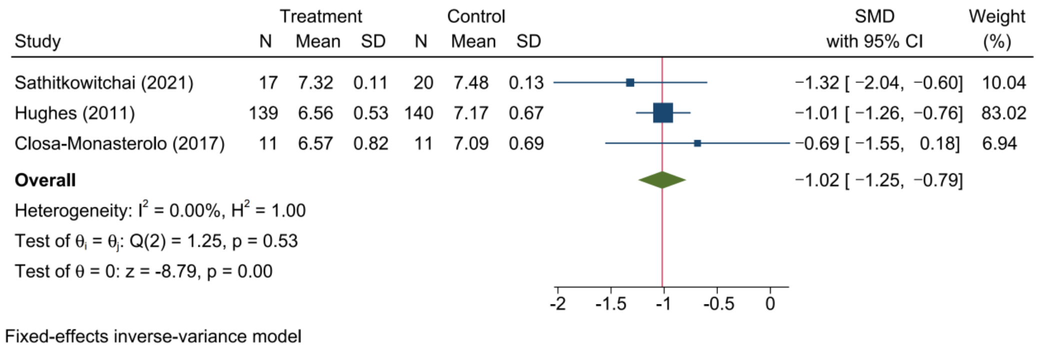

Figure S11. Forrest plot reporting SMD and 95%CI for the effects of NDOs on fecal pH.

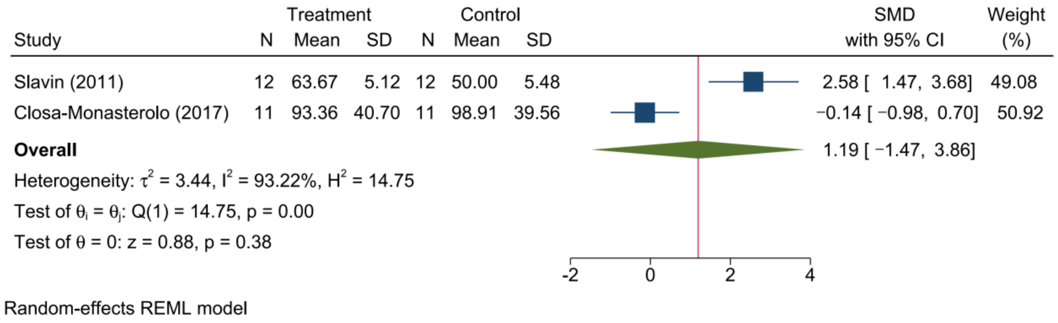

Figure S12. Forrest plot reporting SMD and 95%CI for the effects of NDOs on fecal total SCFA.
